# Further Insights Into the Metabolism of LGD‐4033 in Human Urine. Part 2. A New Minor Metabolite With Antagonistic Activity on the Androgen Receptor Can Indicate Recent Substance Intake

**DOI:** 10.1002/dta.70005

**Published:** 2025-11-19

**Authors:** Yiannis S. Angelis, Panagiotis Sakellariou, Annekathrin M. Keiler, Mario Thevis, Andreas Thomas, Kevin Lam, Gerhard Wolber, Ariadni Vonaparti, Sven Voss, Michael Petrou, Emmanuel N. Pitsinos

**Affiliations:** ^1^ Institute of Biosciences & Applications National Centre for Scientific Research “DEMOKRITOS”, Doping Control Laboratory of Athens, Neratziotissis & Amaryssias Artemidos Str Athens Greece; ^2^ Institute of Biochemistry/Center for Preventive Doping Research German Sport University Cologne Cologne Germany; ^3^ Institute of Doping Analysis and Sports Biochemistry Dresden Kreischa Germany; ^4^ Environmental Monitoring & Endocrinology, Faculty of Biology Technische Universität Dresden Dresden Germany; ^5^ European Monitoring Center for Emerging Doping Agents Cologne Germany; ^6^ Institute of Pharmacy Freie Universität of Berlin Berlin Germany; ^7^ Cyprus Anti‐Doping Authority Makarion Athletic Centre Avenue, Engomi Nicosia Cyprus; ^8^ Institute of Nanoscience and Nanotechnology National Centre for Scientific Research “DEMOKRITOS” Athens Greece; ^9^ Department of Chemistry, National and Kapodistrian University of Athens Athens Greece

**Keywords:** androgen receptor binding, contamination scenarios, doping, human metabolism, LGD‐4033

## Abstract

Using a targeted metabolic investigation approach, a new, previously undescribed metabolite, which is a pyrrole derivative of LGD‐4033, has been detected and coded as **M8**. This metabolite can be detected in postadministration human urine samples up to 6 days after administration. It has also been detected in post‐administration samples, mimicking supplement contamination, after repeated 10 μg doses detectable for ≥ 120 h after administration. Given **M8's** structural similarity to LGD‐4033, its androgen receptor (AR) agonist/antagonist properties were studied using *in silico* molecular docking and functional in vitro AR transactivation assays in the PC3(AR)_2_ cell model, alongside other selected LGD‐4033 metabolites. The results indicate that **M8** can act as a potent AR antagonist, whereas **M2c** was reconfirmed as a potent AR agonist. Therefore, we propose the inclusion of **M2c** in ITP doping control methods, as it could be used as an LGD‐4033 alternative and may be introduced into the black market. Additionally, the detection of **M8**, which is an early‐stage excreted metabolite, is valuable for estimating sample collection time relative to LGD‐4033 intake. When combined with the evaluation of other long‐term metabolites like M5b, M5a, M2c, and M2d, **M8** detection can aid in distinguishing adverse analytical findings, associated abuse through regular dosing, from unintentional doping caused by certain contamination scenarios or abuse through microdosing.

## Graphical abstract

A novel early excreted minor metabolite of LGD‐4033 was detected and coded as M8. Its presence was evaluated as an indicator of recent substance intake versus end‐tail findings. Additionally, the agonist/antagonist properties of M8 and other selected LGD‐4033 metabolites on the androgen receptor (AR) were studied using in silico molecular docking and binding measurements in the PC3(AR)2 cell model, and the results are presented.

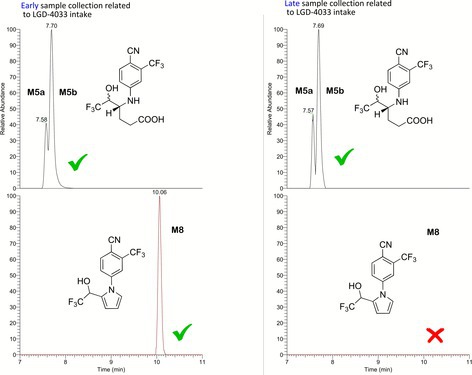

## 1 Introduction

**FIGURE 1:**
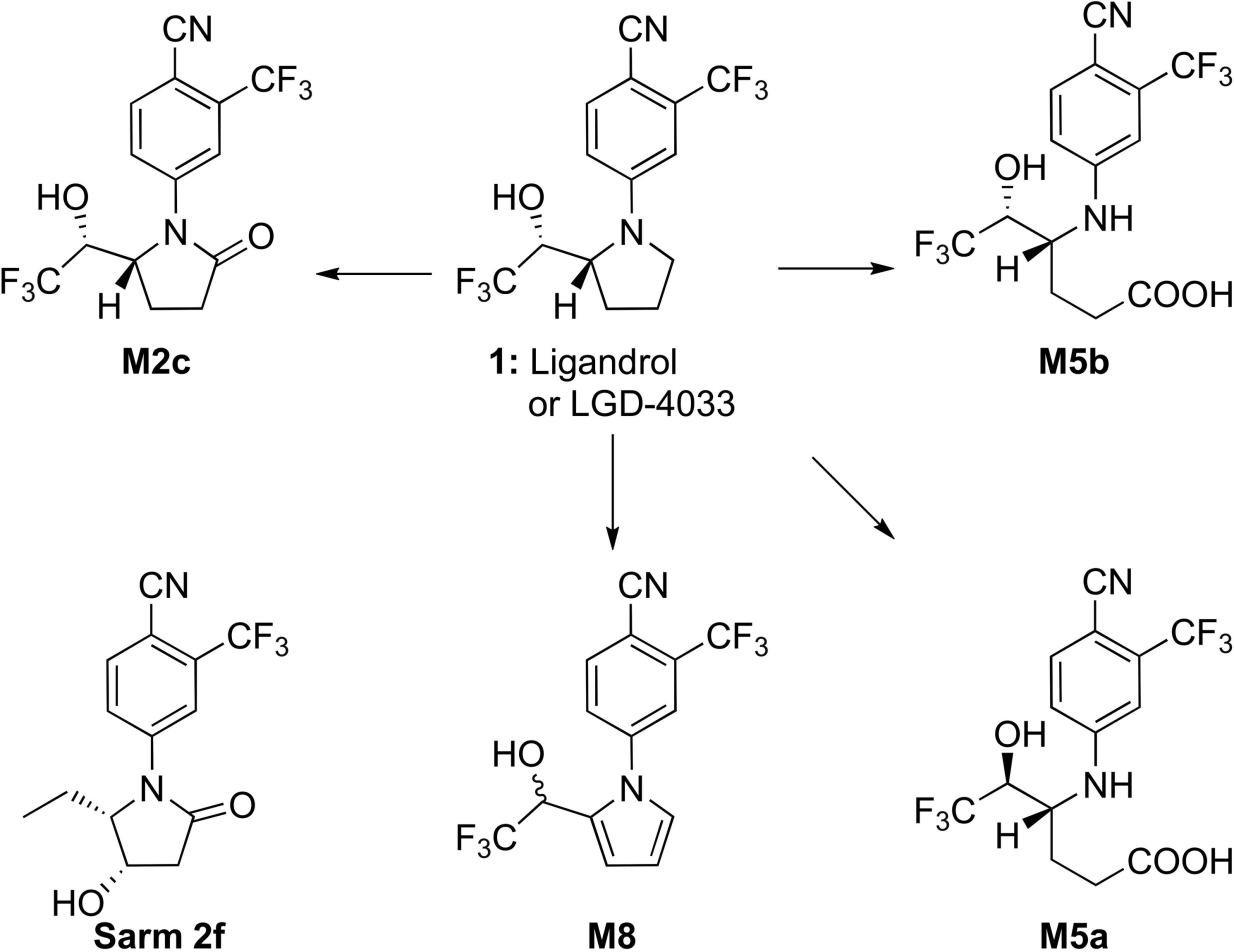
Sarm 2f and selected LGD‐4033 metabolites. The metabolite coding follows that presented by Wagener et al. [13].

In general, the best biomarkers for monitoring a substance's abuse within the context of sports drug testing are not those excreted in higher amounts but rather those excreted for the longest period of time after intake. Hence, ongoing research, which aims to expand current knowledge on the metabolic fate of substances related to doping and considers analytical instrumentation upgrades, is justified [1, 2]. Such research has revealed a number of long‐term metabolites that substantially extend the detection time windows for many substances commonly detected in doping control samples, including stanozolol [3, 4], metandienone [2, 5, 6], dehydrochloromethyltestosterone [7, 8] oxandrolone [9], methenolone [10, 11, 12], and many others. On the other hand, in the last decade, the detection of only long‐term metabolites in doping control urine samples has been associated, in some cases, with the inadvertent exposure to a doping agent. Consequently, adverse analytical findings (AAFs) for several doping substances, including Ligandrol/LGD‐4033 (**1**, Figure 1), during doping control have been challenged [14, 15, 16, 17, 18, 19, 20, 21]. Consumption of contaminated drugs or supplements, or transfer of drug metabolites during interpersonal contact, including intimate moments, has emerged as a possibility [14, 20], where the detection of a long‐term metabolite might be attributed either to the tail‐end excretion of the corresponding substance or to environmental contamination scenarios. The analysis of additional matrices, especially scalp hair, has been proposed as a discrimination tool for contamination [20, 21, 22]. Kintz et al., for example, investigated the detection of Ligandrol in the hair of a subject and were able to detect it at concentrations down to 7 pg./mL [20]. Limitations of such an approach may include known doping practices, such as microdosing [23, 24, 25]. An alternative discrimination approach might be based on the investigation of a more comprehensive metabolic profile of a substance during sports doping control, where long‐term metabolites will be monitored along with early‐stage excreted metabolites to correlate a finding with the time of intake [13]. Wagener et al. detected LGD‐4033 long‐term metabolites in postadministration samples designed to mimic drug contamination scenarios and proposed a metabolite ratio as a discrimination tool between contamination and doping [13].

LGD‐4033 is an extensively metabolized compound in human and other examined species [13, 26, 27, 28, 29, 30, 31, 32, 33, 34, 35] with **M5b** ((4*R*,5*R*)‐4‐{[4‐cyano‐3‐(trifluoromethyl)‐phenyl]amino}‐6,6,6‐trifluoro‐5‐hydroxyhexanoic acid, Figure 1) being the metabolite of choice for monitoring its abuse within the context of human sports drug testing. Following our continuing interest in the structural elucidation of LGD‐4033 metabolites [31, 32], a new, previously undescribed metabolite, namely, a pyrrole derivative **M8** (Figure 1), was detected as a minor metabolite during a targeted metabolic investigation employing a synthetic compound as a standard in the analysis of LGD‐4033 post‐administration human urine samples. The structure of this metabolite along with selected LGD‐4033 metabolites with long‐term potential, namely, **M5a**, **M5b**, and **M2c**, is presented in Figure 1. The pyrrole derivative **M8** can be detected in post‐administration samples, including some post‐administration samples collected after micro‐dosing of LGD‐4033 that mimicked supplement contamination [13]. Interestingly, the pyrrole derivative **M8**, as well as other LGD‐4033 metabolites, such as its pyrrolidinone derivative (coded as **M2c**), exhibit very close structural similarity to the parent LGD‐4033 and retain all the structural features that contribute to the binding and transactivation of LGD‐4033 with the androgen receptor (AR). Hence, the investigation of their binding and transactivation properties for the AR was considered highly relevant in the fight against doping. To this end, the pyrrole derivative **M8**, along with the parent LGD‐4033, its pyrrolidinone derivative **M2c**, and the LGD‐4033 long‐term metabolite **M5b** were selected, and their binding properties for the AR were investigated. Two complementary approaches were used to investigate these properties: direct binding experiments on the AR using the human PC3(AR)_2_ cell model and an in silico molecular docking study in which the binding mode of LGD‐4033 and its metabolites was compared with those of SARM 2f (Figure 1), another anabolic agent that received growing attention in sports drug testing recently [26]. From a metabolic perspective, the identification and characterization of this novel metabolite enhance our current knowledge of the metabolic fate of LGD‐4033, and its presence in a suspicious sample can improve the interpretation of doping control results by indicating that the sample was collected close to the time of exposure to LGD‐4033.

## 2 Materials and Methods

Detailed information, regarding the synthesis of **M8** and the other materials used during this study, is provided in the Supplementary Information. In the same section, appropriate information on the post‐administration samples, the sample preparation procedures, and the LC‐HRMS/MS methods employed, as well as on the AR transactivation assay and the in silico molecular modeling, is also included.

## 3 Results and Discussion

### 3.1 Synthesis and Targeted Investigation of M8 in Postadministration Samples

**FIGURE 2:**
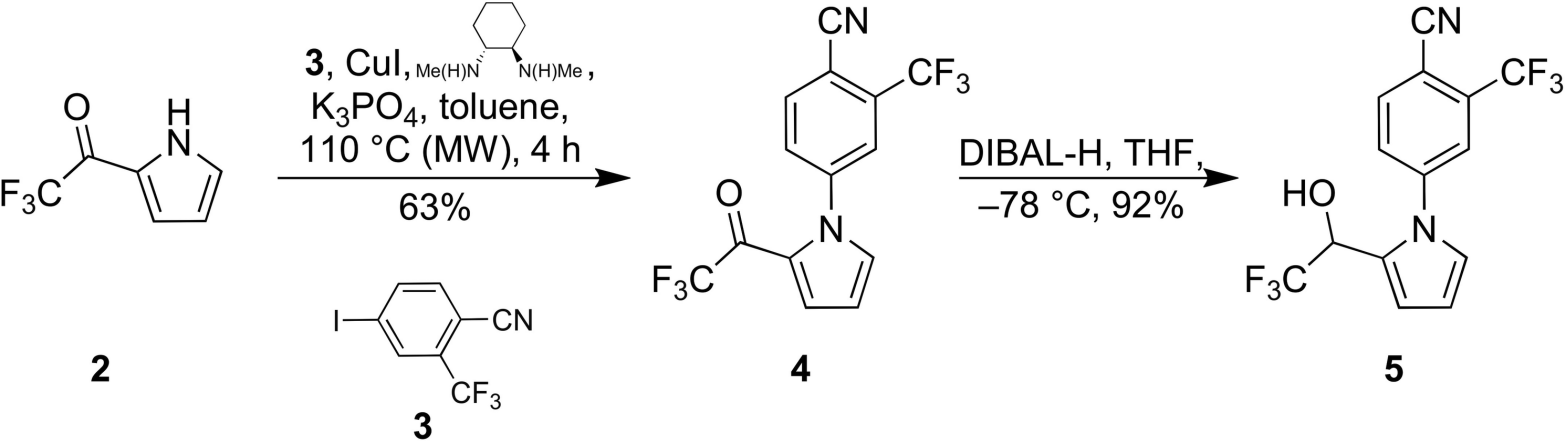
Synthesis of pyrrole derivative **5**.

During initial exploratory synthetic studies [31] toward the originally proposed structure for the main long‐term metabolite of LGD‐4033 [27], pyrrole **5** (Figure 2) was targeted as a potential entry point. To this end, the required 1‐aryl‐2‐trifluoroacetyl‐pyrrole **4** (Figure 2) was secured in fair yield (63%) through coupling of 2‐trifluoroacetyl‐pyrrole (**2**) with 4‐iodo‐2‐(trifluoromethyl)benzonitrile (**3**) under slightly modified Buchwald conditions (5 mol% CuI, 20 mol% *trans*‐*N,N′*‐dimethyl‐cyclohexyl‐1,2‐diamine, 2.1 equiv. K_3_PO_4_, microwave heating at 110 °C, 4 h) [36, 37]. The corresponding 2‐trifluorohydroxyethyl‐pyrrole derivative (**5**), was prepared through reduction (DIBAL‐H) of **4's** carbonyl group.

Further elaboration of **5** to the targeted dihydroxylated metabolite through oxidative dearomatization [38] was not successful. Nonetheless, the availability of the above‐mentioned synthetic intermediates prompted their evaluation under targeted liquid chromatography–high resolution (tandem) mass spectrometry (LC‐HRMS/(MS)) analytical methods as potential, previously unreported, metabolic markers of LGD‐4033. Among them, of particular interest was the pyrrole derivative **5** (Figure 2), because it could plausibly arise metabolically through oxidative aromatization of the LGD‐4033 pyrrolidine ring.

**FIGURE 3:**
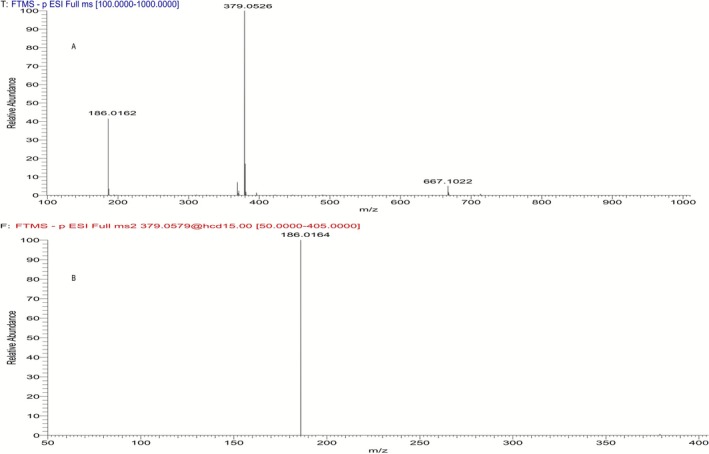
(A) Full scan mass spectrum of pyrrole derivative **5**. (B) Product ion mass spectrum of the precursor ion at *m/z* 379.0526 of the pyrrole derivative **5**.

Two main signals were observed in the full scan HRMS spectrum of the synthesized pyrrole derivative **5** in negative electrospray ionization (ESI) mode (Figure 3A). The main ion at *m/z* 379.0526 can be attributed to its formate adduct (mass error 3 ppm). A second, less abundant ion at *m/z* 186.0162 is attributable to a fragment corresponding to the aryl group of LGD‐4033 with a substitution of the aryl nitrogen by an oxygen (i.e., the anion of 4‐hydroxy‐2‐(trifluoromethyl)benzonitrile) (mass error 1 ppm). A minor signal of 2 M‐H was also observed at *m/z* 667.1022 (mass error 6.6 ppm). Interestingly, only one product ion at *m/z* 186.0164 was observed in the product ion mass spectrum of the precursor ion at *m/z* 379.0526 (Figure 3B).

**FIGURE 4:**
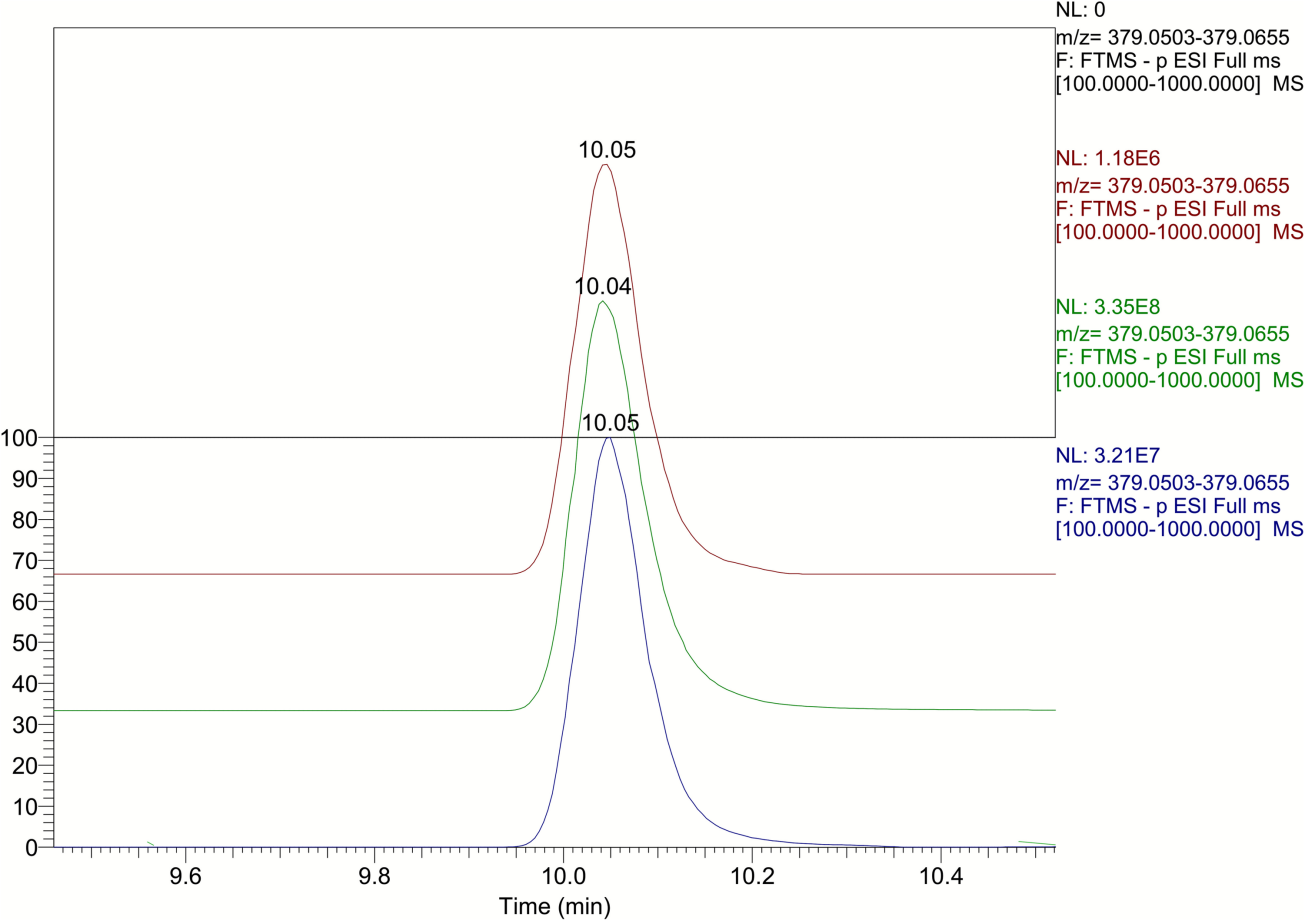
Comparison of extracted ion chromatograms at *m/*z 379.0526 of urine samples collected at 0 h post‐administration (black color), 16 h (blue color), and 6 days (red color) postadministration and of a pure synthetic standard of the pyrrole derivative **5** (green color).

Targeted metabolic investigation for this analyte in post‐administration human urine samples and comparison with a preadministration sample revealed pyrrole **5** as a new, not previously described, metabolite of LGD‐4033 (**M8**). This metabolite is excreted exclusively in the glucuronide fraction and can be detected up to 6 days after administration. A representative comparison of extracted ion chromatograms at *m/z* 379.0526 of postadministration urine samples collected at different time points is presented in Figure 4. To rule out the possibility that this metabolite may originate as a contamination of the supplement used in the excretion study, the supplement was analyzed. Nonetheless, the analyte of interest was not detected in it.

Biosynthetically, this metabolite might arise through double hydroxylation of LGD‐4033 to produce the dihydroxy pyrrolidine that was originally suggested as the main bis‐hydroxylated long‐term metabolite [27]. Such entities have been postulated as intermediates in the Paal–Knorr synthesis of pyrroles through the condensation of 1,4‐dicarbonyl compounds with ammonia, primary amines, or anilines [39]. Consequently, it is reasonable to expect that, upon formation, a dihydroxy pyrrolidine would readily suffer double dehydration to yield pyrrole **5**. An alternative metabolic pathway to **M8** might involve a pyrrolidine ring hydroxylation followed by sulfation and elimination to introduce the first unsaturation. Subsequent introduction of the second double bond should be thermodynamically favored because of the aromatic character of the final metabolic product. However, to date, no sulfated metabolites have been reported for LGD‐4033. Irrespective of its metabolic origin, pyrrole **5** (**M8**) constitutes a novel metabolite that expands our knowledge on the metabolism of LGD‐4033.

**FIGURE 5:**
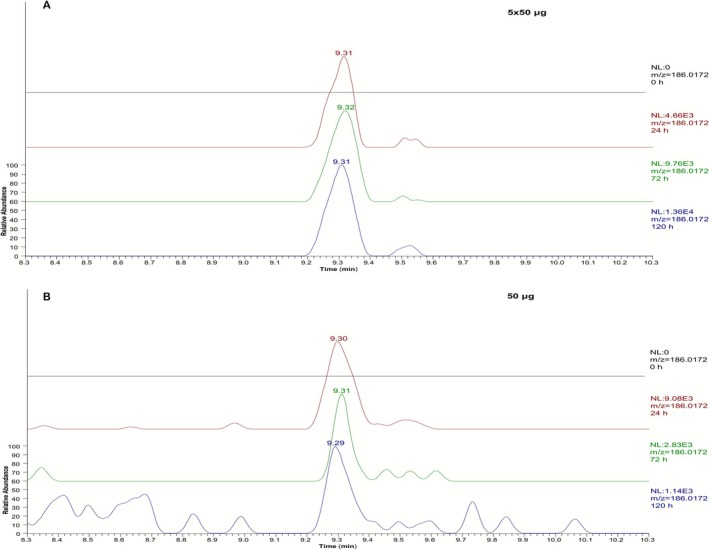
Representative extracted ion chromatograms at *m/z* 186.0172 of preadministration sample (grey) and 24 h (red), 72 h (green), and 120 h (purple) post‐administration samples for (A) the 5 × 50 μg and (B) a single 50 μg dose.

**FIGURE 6:**
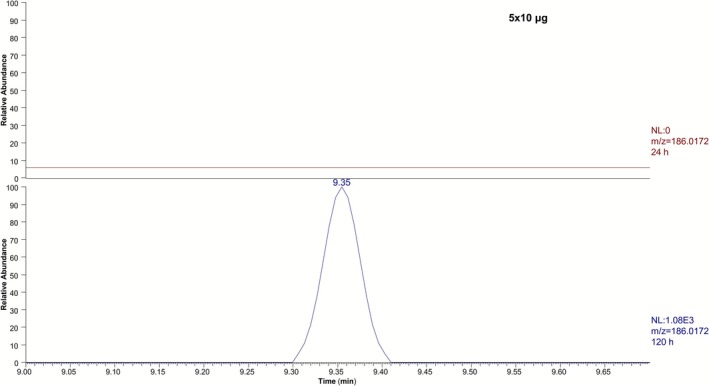
Representative extracted ion chromatograms at *m/z* 186.0172 of 24 h (red) and 120 h (purple) post‐administration samples for the 5 × 10 μg dose.

The analysis of post‐administration samples from the excretion study that mimicked supplement contamination using a selected ion monitoring (SIM) method in the negative ionization mode for the *m/z* 393.0679 (acetate adduct) and *m/z* 186.0172 led to the detection of the pyrrole derivative **5** (**M8**) in low‐dose postadministration samples (Figure 5). The aryl phenol fragment (at *m/z* 186.0172) is much more sensitive than the acetate adduct (at *m/z* 393.0679), which could only be used for the monitoring of the high‐dose administration. The aryl phenol fragment (at *m/z* 186.0172) was detected up to 192 h after administration in the 5 × 50 μg study, up to 120 h after the administration of 50 μg of LGD‐4033, and from 72 to 120 h in the 5 × 10 μg study, indicating a bioaccumulation phenomenon (Figure 6). The new metabolite was not detected upon analysis of the samples from the lower dose administration studies (1, 10, and 5 × 1 μg). The long‐term metabolite was detectable until the end of the excretion in all cases. The samples of the same collection times from a second volunteer, regarding the 5 × 50 μg study, were also analyzed, confirming the detection of **M8** up to 192 h after administration.

Pyrrole derivative **5** (**M8**) constitutes a novel metabolite, expanding the knowledge on the metabolism of LGD‐4033. Even though it is a minor, early‐stage excreted metabolite, its presence in a sample could provide valuable information to doping control laboratories and testing authorities regarding the time of LGD‐4033 intake. Additionally, as environmental contamination is getting increasingly significant in the antidoping field and samples with very low concentrations are reported as AAFs, pyrrole **5** (**M8**) could emerge as an important metabolic marker for distinguishing between these cases. Furthermore, monitoring this new metabolite, along with the evaluation of the long‐term metabolites (**M5a** and **M5b**) and other late‐stage excreted metabolites (e.g., **M2c**), could assist testing authorities in distinguishing between findings of late‐stage excreted metabolites associated with abuse through regular dosing and cases of unintentional doping caused by supplements contaminated with LGD‐4033 or of abuse through microdosing, ultimately protecting clean athletes in the antidoping context. Consequently, future analytical studies related to the excretion of pyrrole **5** (**M8**) after micro‐dose administration of LGD‐4033 are warranted.

### 3.2 Binding and Transactivation Properties of LGD‐4033, M2c, M5b, and M8

#### 3.2.1 AR Activation Properties in the Human PC3(AR)_2_ Cell Model

**FIGURE 7:**
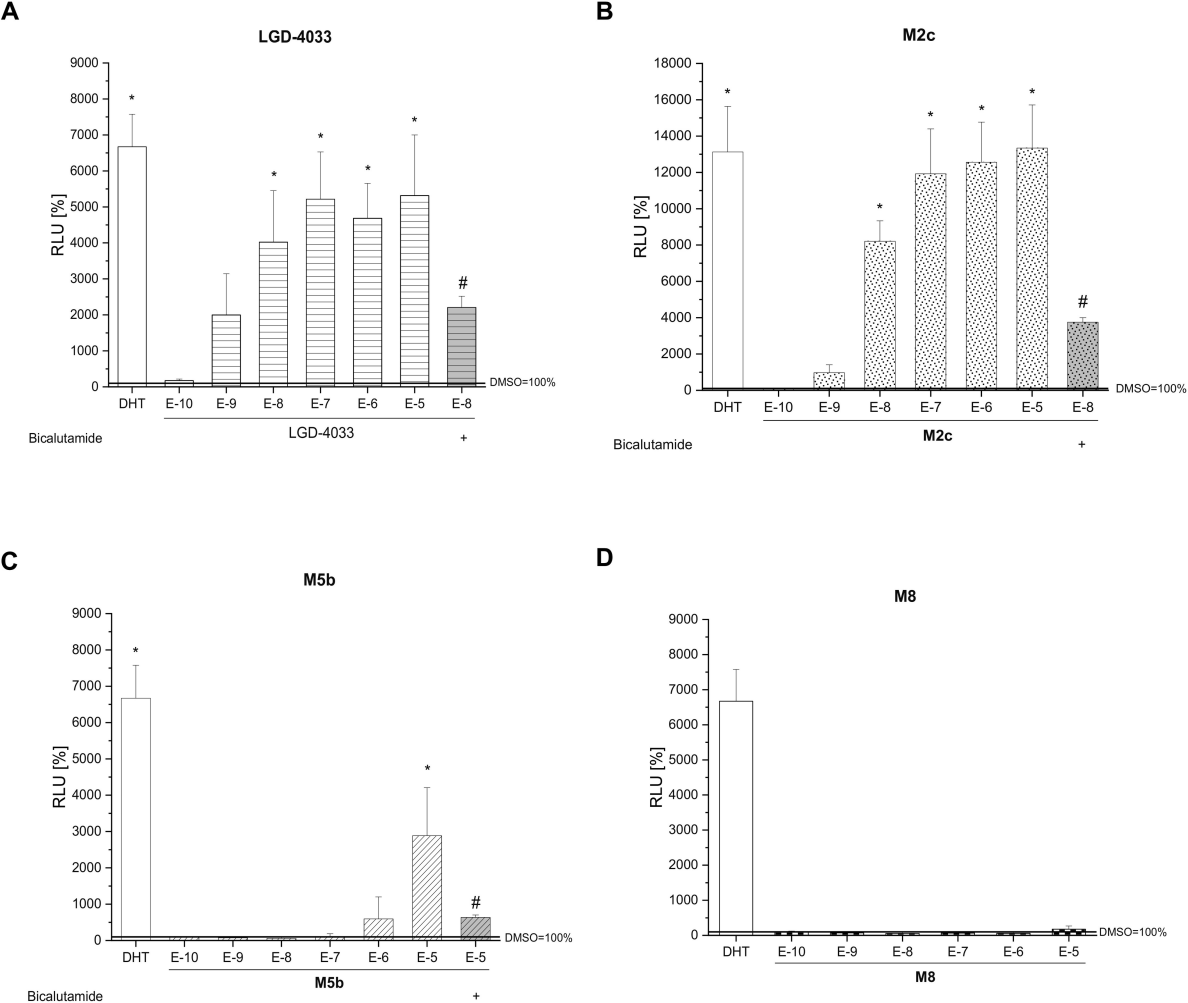
Agonistic activities. Dose response curves are shown for (A) LGD‐4033 (**1**), (B) **M2c**, (C) **M5b,** and (D) **M8** compared to 10^−9^ M DHT as positive control. Reporter gene expression in PC3(AR)2 cells induced by LGD‐4033, **M2c**, or **M5b** could be antagonized by respective co‐incubation with the AR antagonist bicalutamide (5 × 10^−7^ M). Luminescence is shown relative to the solvent control dimethyl sulfoxide (DMSO) (relative luminescence units [RLU] = 100%). * denotes statistically significant differences compared to the solvent control DMSO; # denotes statistically significant differences of the co‐treatment with bicalutamide compared to the respective single treatment with test compounds **1**, **M5b**, or **M2c** (*p* < 0.05).

**FIGURE 8:**
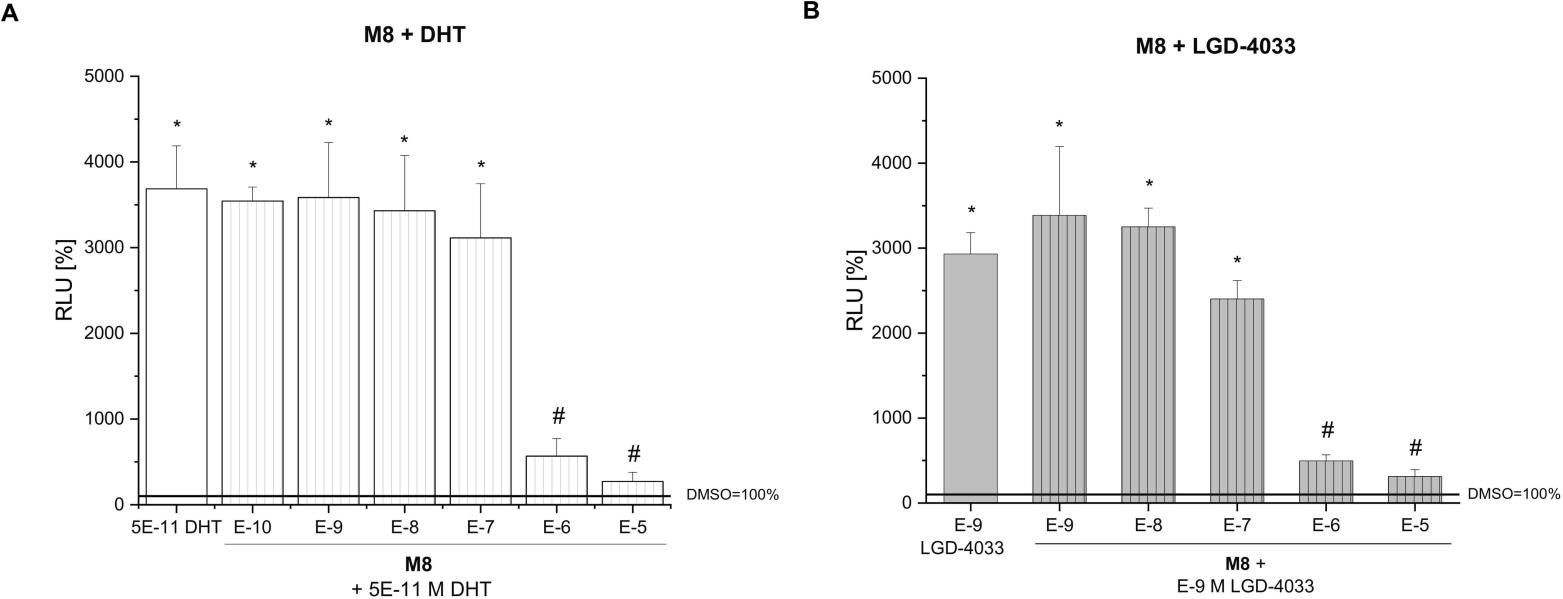
Antagonistic properties of **M8**. The antagonistic activity of **M8** on (A) the AR activation by DHT and on (B) the AR activation by LGD‐4033 is shown in a dose‐dependent manner. Luminescence is shown relative to the solvent control DMSO (RLU = 100%). * denotes statistically significant differences compared to the solvent control DMSO. # denotes statistically significant differences of the co‐treatment with bicalutamide compared to the respective single treatment with (*p* < 0.05).

LGD‐4033 and metabolite **M2c** induced luciferase expression in the human prostate cell line in a dose‐dependent way (Figure 7A,B). This illustrates the AR binding and activating properties of both compounds, as the AR antagonist bicalutamide was able to significantly inhibit the stimulated reporter gene activity. The pyrrolidinone derivative **M2c** possesses as strong agonist properties as LGD‐4033 (EC_50_ = 6.311 E−9 and EC_50_ = 1.406 E−9 respectively), an intriguing finding that is in accord with previous reports [40]. In contrast, the LGD‐4033 long‐term metabolite **M5b** showed rather weak agonistic activities in the bioassay, as it induced statistically significant relative luminescence solely at the highest dose tested (Figure 7C). In contrast, the newly detected LGD‐4033 pyrrole derivative **M8** did not induce AR‐dependent reporter gene expression in the PC3(AR)2 cells at any of the concentrations tested (Figure 7D). While agonist properties were lacking, **M8** showed antagonistic activities by inhibiting both dihydrotestosterone‐ (DHT) and LGD‐4033–induced stimulation of luciferase gene expression (Figure 8).

#### 3.2.2 In Silico Molecular Modeling

**FIGURE 9:**
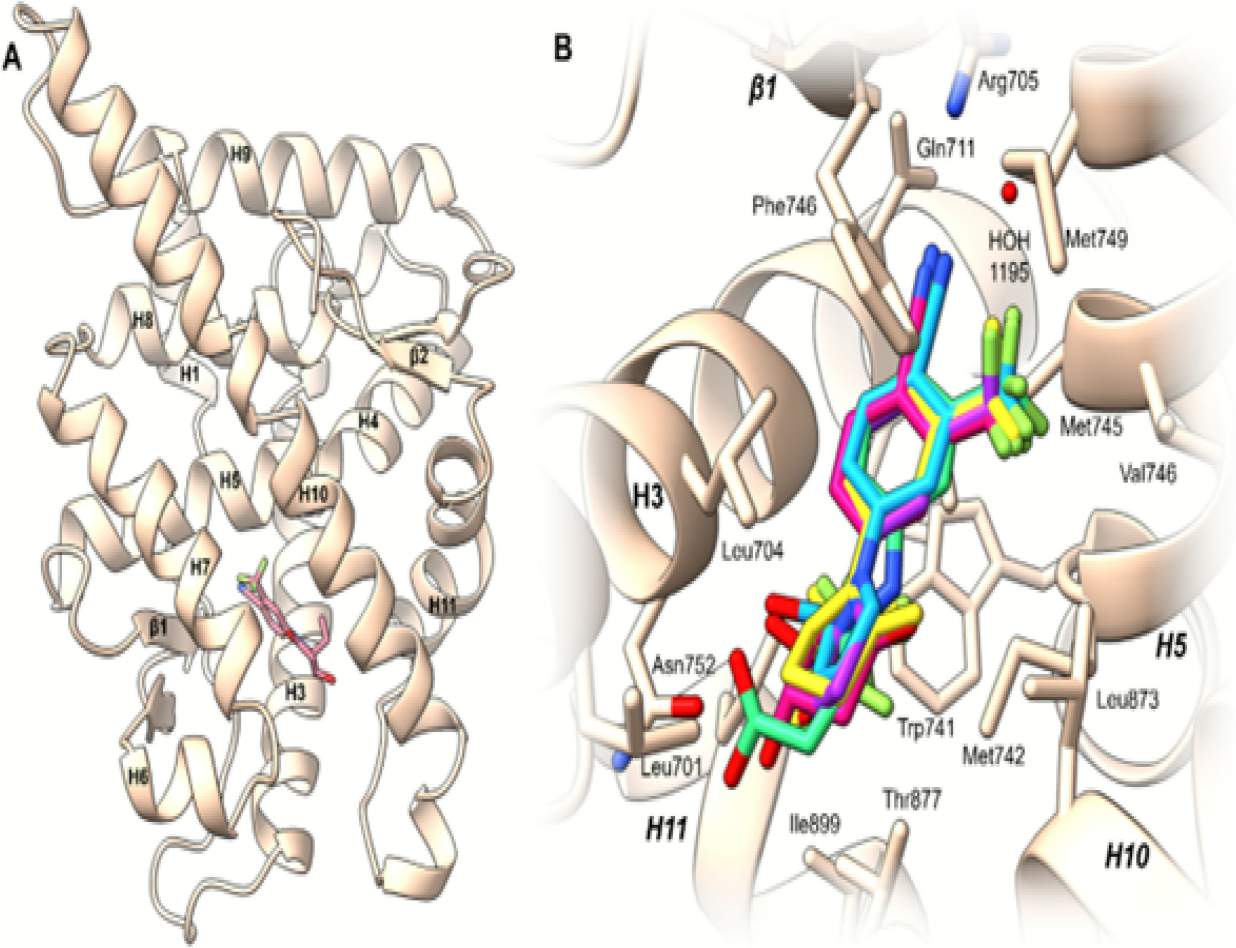
(A) Structure of AR co‐crystallized with SARM‐2f located in the LBS shaped by H3, H5, H7, H10, H11, and β1. (B) SARM‐2f (red) in LBS superposed with the metabolites **M5b** (green), **M2c** (yellow), **(*s*)‐M8** (cyan), and **(*R*)‐M8** (purple).

AR's ligand binding site (LBS) is located in a small hydrophobic cavity formed by helices H3, H5, H7, H10, H11, and beta‐sheet β1. Ligands that bind to the LBS typically form hydrogen bonds with Asn705 and Arg752 [41]. The binding mode of SARM‐2f (Figure 1), which is structurally similar to LGD‐4033 and its metabolites, also follows this pattern (Figure 9A) [42]. Molecular docking studies were conducted to investigate whether LGD‐4033 and its metabolites, namely, **M2c**, **M5b**, and **M8**, can achieve the same binding mode as SARM‐2f in AR.

**FIGURE 10:**
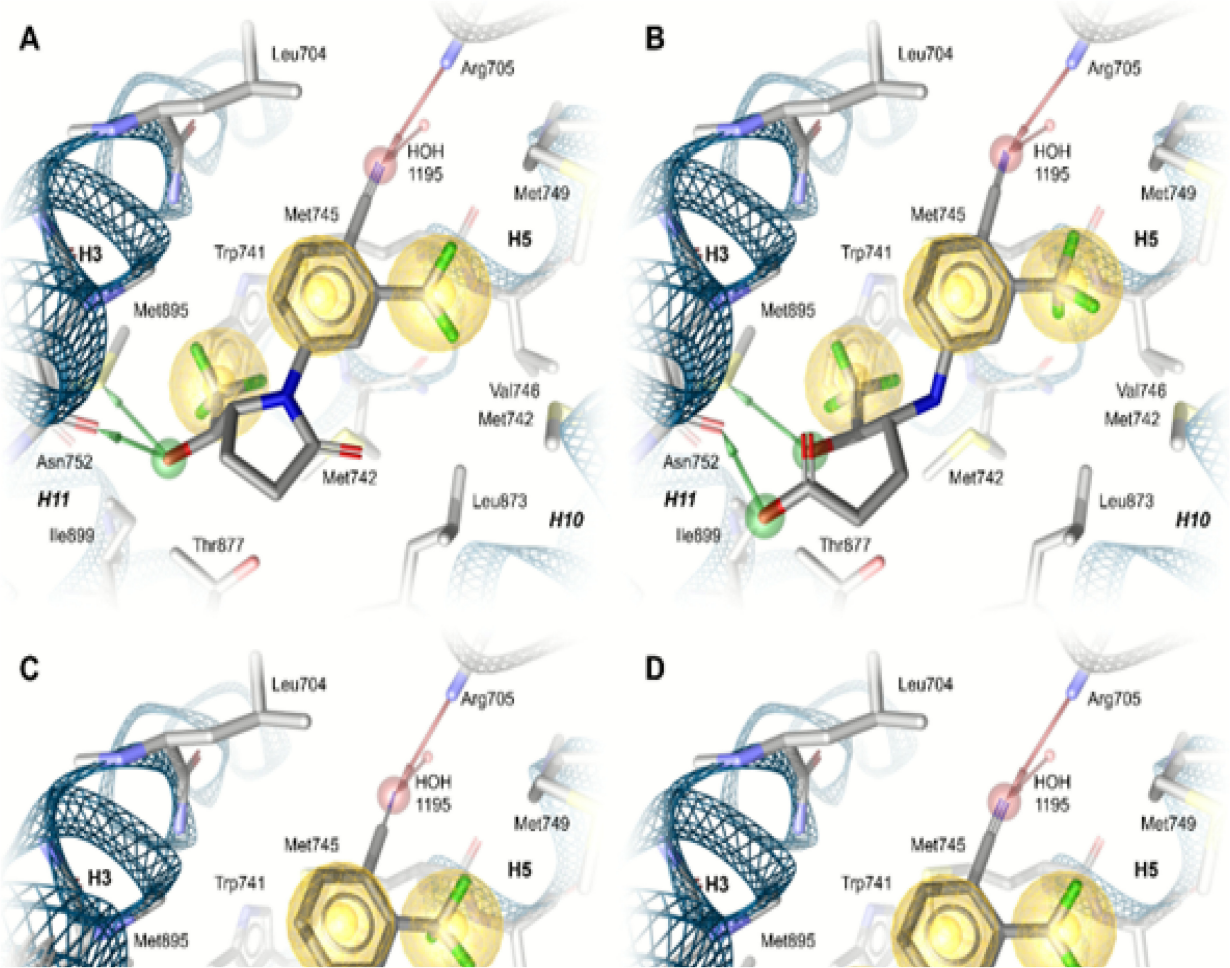
Binding hypotheses of (A) **M2c**, (B) **M5b**, (C) (*S*)*‐* **M8**, and (D) (*R*)‐**M8** in AR. Red arrows depict hydrogen acceptor bonds, green arrows show hydrogen donor bonds, and yellow spheres show hydrophobic contacts formed with surrounding hydrophobic residues.

All binding modes show favorable shape similarity with SARM‐2f in AR (Figure 9B). In each binding mode, hydrogen acceptor bonds are formed between the nitrile moiety and HOH1195 and Arg705, while the phenyl ring and both trifluoroalkyl moieties form hydrophobic contacts with the surrounding residues (Figure 10). Hydrogen donor bonds with Met895 and Asn752 are also found for each binding mode. While **M2c** and both isomers of **M8** form the hydrogen donor bond with Asn752 through their OH moiety, the carboxylic acid is responsible for forming this hydrogen bond for the uncharged state of **M5b**. Unique pharmacophore features are also found for **M8**, where the pyrrole ring forms an additional hydrophobic contact, and specifically for (*S*)‐**M8**, which forms a weak hydrogen acceptor bond between the hydroxytrifluoroethyl chain and Thr877.

For the deprotonated state of **M5b**, no binding hypothesis could be found in which the charged carboxylate and OH‐moiety perform interactions simultaneously. This strongly suggests that the deprotonated state should be inactive. Additionally, the increased amount of rotational bonds in **M5b**'s carbon chain, which carries the OH‐moiety and carboxylic acid, results in an entropic penalty, which decreases its overall binding affinity [42]. We suspect that both the flexible carbon chain and the deprotonated ligand contribute to the low activity of **M5b**.

## 4 Conclusions

The synthesized pyrrole derivative **5** (Figure 1) corresponds to a new, previously undescribed metabolite of LGD‐4033, which was assigned the code **M8.** It can be detected in postadministration samples up to 6 days after administration as well as in post‐administration samples that mimic supplement contamination up to multiple doses of 10 μg for at least 120 h after administration. Its presence in a sample could provide valuable information and, along with the evaluation of long‐term metabolites, may aid the estimation of the timing of LGD‐4033 intake. This distinction is crucial for differentiating intentional misuse through regular dosing from unintentional doping caused by several contamination scenarios or intentional LGD‐4033 microdosing as a possible doping practice. Additionally, due to its close structural similarity to the parent LGD‐4033, its binding and transactivation properties toward the AR were studied, along with other selected LGD‐4033 metabolites. **M8** exhibited antagonistic action to the AR, while strong agonist properties were observed for **M2c**. Hence, since it could be used as an alternative to LGD‐4033 and might, at some point, be introduced into the black market, its inclusion in the initial testing procedures (ITP) for doping control is warranted as a proactive measure.

## Author Contributions

Yiannis S. Angelis: conceptualization, funding, data acquisition, data curation, manuscript writing, manuscript editing. Panagiotis Sakellariou: data acquisition, data curation, manuscript writing, manuscript editing. Annekathrin Keiler: data acquisition, data curation, manuscript editing. Mario Thevis: funding, manuscript editing. Andreas Thomas: data curation, manuscript editing. Kevin Lam: data acquisition, data curation, manuscript editing. Gerhard Wolber: data acquisition, data curation, manuscript editing. Ariadni Vonaparti: data acquisition, data curation, manuscript editing. Sven Voss: funding, manuscript editing. Michael Petrou: funding, manuscript editing. Emmanuel N. Pitsinos: funding, conceptualization, synthesis of compounds, data curation, manuscript writing, manuscript editing.

## Conflicts of Interest

The authors declare no conflicts of interest.

## Funding

This work was supported by the World Anti‐Doping Agency (21A17EP).

## Supporting information


**Data S1:** Supplementary Information.

## Data Availability

The data that support the findings of this study are available from the corresponding author upon reasonable request.
