# Supplementary material for: Further Insights Into the Metabolism of LGD‐4033 in Human Urine. Part 2. A New Minor Metabolite With Antagonistic Activity on the Androgen Receptor Can Indicate Recent Substance Intake
Source: Drug Test Anal. 2025 Nov 19;18(1):159–69. doi: 10.1002/dta.70005 (PMC12796561; doi:10.1002/dta.70005)
Supplement: Supplementary file 1 — Data S1: Supplementary Information. [file DTA-18-159-s001.docx]

**Supplementary Information**

**Further Insights into the Metabolism of LGD-4033 in Human Urine Part 2. A New Minor Metabolite with Antagonistic Activity on the Androgen Receptor Can Indicate Recent Substance Intake**

Yiannis S. Angelis^[a]^*, Panagiotis Sakellariou^[b]^, Annekathrin M. Keiler^[c,d]*^ Mario Thevis^[b,e]^, Andreas Thomas^[b]^, Kevin Lam^[f]^, Gerhard Wolber^[f]^, Ariadni Vonaparti^[c]^, Sven Voss^[c]^, Michael Petrou^[g]^, Emmanuel N. Pitsinos^[h,i].^

**1 Materials**

All reagents were purchased at the highest commercial quality and used without further purification, unless otherwise stated. LGD-4033 (CAS Number 1165910-22-4) and *epi*-LGD-4033 (CAS Number 1165910-23-5) were procured from Toronto Research Chemicals.

Stock solutions of the synthesized reference materials were prepared in methanol and stored at −20 °C. Ethyl acetate were of analytical grade and obtained from Labscan (Dublin, Ireland). Dipotassium hydrogen phosphate (K_2_HPO_4_), potassium dihydrogen phosphate (KH_2_PO_4_), disodium carbonate (Na_2_CO_3_), sodium hydrogen carbonate (NaHCO_3_) and sodium sulfate (Na_2_SO_4_) were purchased from Panreac (Barcelona, Spain). Disodium hydrogen phosphate monohydrate (Na_2_HPO_4_**^.^**H_2_O), potassium carbonate (K_2_CO_3_) and potassium bicarbonate (KHCO_3_) were obtained from VWR chemicals (Radnor, USA). Acetonitrile (ACN) for mobile phase preparation was of LC-MS grade and purchased from Riedel-de Haen (Seelze, Germany). Acetic acid, formic acid and sodium dihydrogen phosphate (NaH2PO4) were purchased from Merck (Darmstadt, Germany) and ammonium formate from Acros Organics. HPLC-grade water was obtained from purifying water in a filtration system (Millipore, Billerica, MA, USA). β-glucuronidase from *Escherichia coli* was obtained from Sigma-Aldrich (Steinheim, Germany).

For the *in vitro* assays, Sigma-Aldrich (Munich, Germany) provided dihydrotestosterone (DHT, purity ≥ 97.5 %) and bicalutamide (purity ≥ 98 %). Carl Roth (Karlsruhe, Germany) provided dimethyl sulfoxide (DMSO, purity ≥ 99.5 %). Dulbecco's Modified Eagles Medium (DMEM/F12), fetal bovine serum (FBS) and penicillin/streptomycin (P/S) were supplied by BioWest (Nuaillé, France). Qiagen (Hilden, Germany) provided Attractene Transfection Reagent. G418 was supplied by Calbiochem, Merck (Darmstadt, Germany). Promega (Mannheim, Germany) provided the luciferase assay system. AppliChem (Darmstadt, Germany) provided bovine serum albumin fraction V (BSA). Dr. Aria Baniahmad (Institute for Human Genetics, University Hospital Jena) kindly provided human PC3(AR)_2_ cells and the reporter plasmid encoding the luciferase gene controlled by the mouse mammary tumor virus long repeat promotor (mmTV-luc).

**2 Synthesis of compounds**

**2.1 Synthesis methods**

All reactions were carried out under a dry argon atmosphere with anhydrous solvents (freshly distilled over the appropriate desiccant or dried over 3 Å molecular sieves) under anhydrous conditions, unless otherwise noted. Reactions requiring anhydrous conditions were carried out in oven dried (120 °C, 24 h) or flame dried (vacuum < 0.5 Torr) glassware. All reactions were magnetically stirred with Teflon stir bars, and temperatures were measured externally. All reactions were monitored by Thin Layer Chromatography (TLC) carried out on 0.25 mm E. Merck silica gel plates (60F254). UV light (254 nm) was used for visualization and an acidified ethanolic solution of *p*-anisaldehyde or an acidified aqueous solution of ceric ammonium molybdate and heat were used as developing agents. Compounds were purified on a Biotage^®^ Selekt Enkel Automated flash chromatography unit, equipped with the Spektra software and employing Biotage^®^ Sfär Duo silica columns, or by manual flash column chromatography, employing E. Merck silica gel (60 Å, particle size 0.040–0.063 mm) or Acros Organics silica gel (60 Å, particle size 0.035–0.070 mm). Optical rotations were recorded using a Perkin-Elmer 241 polarimeter at the sodium D line (589 nm) using a 10 cm path-length cell in the solvent and concentration indicated. Infrared (IR) spectra were recorded on a Nicolet 6700 FT-IR spectrometer. Nuclear Magnetic Resonance (NMR) spectra were recorded using a Bruker Avance DRX 500 MHz or a Bruker Avance III 250 MHz instrument and were calibrated using as internal reference the residual nondeuterated solvent for ^1^H-NMR and the deuterated solvent for ^13^C-NMR, respectively (e.g., CDCl_3_: *δ*_H_ = 7.26 ppm, *δ*_C_ = 77.16 ppm; CD_3_OD: *δ*_H_ = 3.31 ppm, *δ*_C_ = 49.00 ppm). Multiplicities are designated as singlet (s), doublet (d), triplet (t), quartet (q), quintet (quint.) or multiplet (m). Broad or obscured peaks are indicated as “br” or “obs”, respectively. To facilitate NMR spectra comparisons, the established LGD-4033 skeleton numbering was used for all compounds when assigning signals. High resolution mass spectra (HRMS) were acquired on a LC qExactive plus HRMS (Thermo Scientific, Bremen, Germany) instrument.

**2.2 Synthetic procedures and characterization data**

**4-[2-(2,2,2-trifluoroacetyl)-1*H*-pyrrol-1-yl]-2-(trifluoro­methyl)­benzonitrile** (**13**): Under an atmosphere of argon, 2-trifluoroacetyl-pyrrole (**11**; 267 mg, 1.64 mmol), finely ground and free flowing tribasic potassium phosphate (806 mg, 3.80 mmol), and 4-iodo-2-(trifluoromethyl)benzonitrile (**12**; 730 mg, 2.46 mmol) were added in a microwave vial, fitted with a rubber septum and equipped with a stirring bar. The vessel was evacuated and back-filled with argon four times. Then, *trans*-*N,N’*-dimethyl-cyclohexyl-1,2-diamine (0.05 mL, 0.4 mmol) and anhydrous and degassed toluene (0.8 mL) were introduced and the mixture was sonicated for 5 min at ambient temperature under a stream of argon. CuI (17 mg, 89 µmol) was added, the rubber septum was removed, the vessel was sealed under a stream of argon, and subjected to microwave irradiation (300 W). The maximum temperature was set to 110 °C with concomitant stirring and air-cooling of the reaction mixture. Initially, pressure build-up to ~35 Psi and stabilized to ~10 Psi after 4 h. The vessel was removed from the microwave reactor and its contents was transferred with the aid of ethyl acetate to a round-bottom flask charged with silica gel (10 g). The resulting slurry was filtered through a short pad of silica gel. The filtrates were concentrated to dryness under reduced pressure and the residue thus obtained was purified chromatographically (silica gel, *n*-hexane/EtOAc 95:5 to 6:4) to provide *N*-aryl pyrrole **13** (341.2 mg, 1.03 mmol; 62.6%) as an off-white solid.

***R_f_*** *=* 0.10 (silica gel, *n*-hexane/EtOAc 8:2); **IR** (film) *ν*_max_ = 3335, 3092, 2924, 2236, 1670, 1615, 1532, 1503, 1457, 1417, 1381, 1311, 1291, 1270, 1241, 1211, 1179, 1137, 1110, 1054, 1022, 918, 873, 856, 849, 768, 757, 734, 684, 644, 600, 565 cm^–1^; **^1^H NMR** (500 MHz, CDCl_3_): *δ* = 7.94 (d, *J* = 8.3 Hz, 1 H, H-6), 7.73 (s, 1 H, H-3), 7.62 (d, *J* = 8.3 Hz, 1 H, H-5), 7.48 (s, 1 H, H-11), 7.22 (s, 1 H, H-9), 6.57 (s, 1 H, H-10) ppm; **^13^C NMR** (125 MHz, CDCl_3_): *δ* = 169.9 (q, *J* = 36.5 Hz, C-12), 143.3 (C-4), 135.6 (C-6), 134.4 (C-9), 134.1 (q, *J* = 33.5 Hz, C-2), 130.1 (C-5), 126.2 (q, *J* = 3.7 Hz, C-11), 125.4 (C-8), 124.9 (q, *J* = 4.7 Hz, C-3), 121.9 (q, *J* = 274.3 Hz, C-16), 116.8 (q, *J* = 290.3 Hz, C-14), 114.7 (C-15), 113.0 (C-10), 110.2 (q, *J* = 2.2 Hz, C-1) ppm; **HRMS** (ESI −): *m/z* calculated for C_15_H_7_F_6_N_2_O_3_^−^ [M+HCOO]^−^: 377.0366, found 377.0369.

**4-[2-(2,2,2-trifluoro-1-hydroxyethyl)-1*H*-pyrrol-1-yl]-2-(tri­fluo­ro­methyl)­benzonitrile** (**10**): To a stirred solution of pyrrole **13** (281.3 mg, 846.8 µmol) in THF (10 mL) cooled to −78 °C was added dropwise 1.0 M solution of DIBAL-H in hexane (1.9 mL, 1.9 mmol). The bath temperature was allowed to gradually warm up to −50 °C over 2 h, at which point TLC analysis indicated complete consumption of the starting material. Excess reagent was quenched by dropwise addition of MeOH (2 mL). The cooling bath was removed, a solution of sodium potassium tartrate tetrahydrate (4.3 g) in water (15 mL) was added to the reaction mixture and stirring was continued at ambient temperature for 1 h. The clear mixture was extracted with ethyl acetate and the organic extracts were washed with brine, dried over Na_2_SO_4_, and concentrated under reduced pressure. Chromatographic purification (silica gel, *n*-hexane/dichloromethane 8:2 to 0:1) of the residue thus obtained provided alcohol **10** (260.6 mg, 779.7 µmol; 92.1% yield) as colourless oil.

***R_f_*** *=* 0.09 (silica gel, CH_2_Cl_2_/*n*-hexane 3:1); **IR** (film) *ν*_max_ = 3423, 2237, 1615, 1507, 1468, 1446, 1345, 1300, 1276, 1180, 1141, 1102, 1055, 997, 902, 857, 786, 727, 707, 684, 660, 564 cm^–1^; **^1^H NMR** (500 MHz, CDCl_3_): *δ* = 7.98 (d, *J* = 8.3 Hz, 1 H, H-6), 7.91 (s, 1 H, H-3), 7.81 (d, *J* = 8.2 Hz, 1 H, H-5), 6.95 (s, 1 H, H-11), 6.69 (s, 1 H, H-9), 6.42 (t, *J* = 3.2 Hz, 1 H, H-10), 4.79 (q, *J* = 6.7 Hz, 1 H, H-12), 2.64 (br s, O*H*) ppm; **^13^C NMR** (125 MHz, CDCl_3_): *δ* = 143.1 (C-4), 136.3 (C-6), 134.8 (q, *J* = 33.5 Hz, C-2), 129.1 (C-5), 126.4 (C-8), 124.5 (C-11), 124.3 (q, *J* = 4.7 Hz, C-3), 124.2 (q, *J* = 281.8 Hz, C-16), 121.9 (q, *J* = 274.5 Hz, C-14), 114.8 (C-15), 112.8 (C-9), 111.3 (C-10), 109.2 (C-1), 65.5 (q, *J* = 33.8 Hz, C-12) ppm; **HRMS** (ESI −): *m/z* calculated for C_15_H_9_F_6_N_2_O_3_^−^ [M+HCOO]^−^: 379.0523, found 379.0527.

**3 Samples**

**3.1 Excretion studies**

**3.1.1 High dose administration study**

Urine samples obtained in a previous administration study [1], which was approved by the National Bioethics Committee of Cyprus (Decision number: EEBK 21.1.01.03/21.04.2017) were used in the present study with the approval of the Bioethics Committee. In more detail, urine samples were obtained after the administration of the supplement Ligandrol (purchased from Neobolics, Montreal, Canada) to one healthy, human male volunteer (Caucasian, 48 years old, 60 kg): one capsule × 10 mg of LGD-4033. The urine samples were collected before (0 h) and after administration up to 494 hours (almost 21 days). The exact time intervals (h) were as follows: 0, 2, 4, 4.5, 8, 10.5, 12, 15, 20, 24, 36, 48, 60, 72, 84, 96, 108, 120, 135, 145.5, 156.5, 168, 180, 193, 204, 216, 228, 240, 252, 264, 279, 288, 300, 312, 324, 337, 349, 361, 373, 385, 397, 409, 420.5, 432, 445, 457, 468.5, 481, and 494. All collected samples were kept frozen at -20 °C until analysis.

**3.1.2 Low dose administration study**

Urine samples from a previous administration study were used [2]. A stock solution of LGD-4033 (Selleckchem), 1 mg/mL in ethanol, was prepared and the corresponding amount for a total of 1, 10 or 50 µg was added to 120 mL of drinking yoghurt. For the single-dose administration study, the spiked yoghurt was ingested by healthy male volunteers. For the multi-dose application study, one spiked yoghurt was consumed on five consecutive days every morning. The urine samples were collected before (0 h) and after administration at specific time points until no analytes related to LGD-4033 were traceable. For this project, selected samples before (0 h) and at 24, 48, 72, 96, 120, 144 and 192 h after administration from one volunteer, regarding both the single- and multi-dose administration study, were analyzed. Informed written consent of the volunteer and approval from the responsible ethical committee were obtained before the start of the study. All collected samples were kept frozen at -20°C until analysis.

**3.2 Sample preparation procedures**

**3.2.1 Laboratory 1**

For the extraction of LGD-4033 metabolites a mixture of internal standards (ISTDs) including dexamethasone-d4 (30 ng/mL), ephedrine-d3 (50 ng/mL), fluticasone propionate-d5 (30 ng/mL), formoterol-d6 (20 ng/mL), furosemide-d5 (200 ng/mL), nalbuphine (25 ng/mL), propranolol-d7 (50 ng/mL), testosterone-d3 (10 ng/mL), and trans-11Nor-Δ9-THC-COOH-d3 (10 ng/mL) was added to 3 mL of urine samples as ISTD. The sample pH was adjusted to 7.0 using phosphate buffer 1 M and then 30 μL β-glucuronidase from *E. Coli* were added. Urine samples were incubated for 1.5 h at 55 ^o^C. Following hydrolysis, urine pH was adjusted to 9.5 with NaHCO_3_:Na_2_CO_3_ (10:1, w/w) solid buffer and hydrolates were extracted with 5 mL of ethyl acetate after the addition of 3 g of anhydrous Na_2_SO_4_ for salting out. Samples were centrifuged and then the organic layer was transferred to glass tubes, the mixture was evaporated to dryness under a stream of nitrogen at 60 ^o^C. Dry residues were reconstituted with 100 μL of a 50 % mixture of ACN in water and transferred to 1.5 mL Eppendorf tubes. Then, 100 μL of 5 mM ammonium formate in 0.02 % formic acid were added, and samples were centrifuged for 10 min at 10,000 rpm. After the centrifuge, 150 μL were transferred to vials with inserts. Twenty microliters of the sample were injected in LC-HRMS/MS without any further purification.

**3.2.2 Laboratory 2**

To 1.5 mL of urine, 10 µL of ISTD (S-24, 10 ng/mL in ACN), 1 mL of sodium phosphate buffer (0.8 M, pH 7.0) and 50 µL of β-glucuronidase from *E. coli* were added before incubating at 50 °C for 1.5 h. Subsequently, a liquid-liquid extraction (LLE) was conducted. For this, the pH was adjusted to 9.6 by adding 500 µL of potassium carbonate buffer (20 %, pH 9.6). Then, the samples were extracted with 4 mL of ethyl acetate by adding 2 g of sodium sulfate to promote salting out and by shaking for 20 min. After centrifugation (2,000 rpm, 10 min), the organic layer was evaporated to dryness under a stream of nitrogen at 50 °C. The dry extracts were reconstituted with 70 μL of ACN, the samples were transferred to vials with inserts and 30 μL of H_2_O were added. Aliquots of 20 μL were analyzed by LC–HRMS/MS.

**4 LC-HRMS/MS analysis**

**4.1 Laboratory 1**

A Dionex UHPLC system (Thermo Scientific, Bremen, Germany) was used for the chromatographic separation. The system consisted of a vacuum degasser, a high-pressure binary pump, an autosampler with a temperature-controlled sample tray set at 7 °C, and a column oven set at 30 °C. Chromatographic separation was performed at 30 °C using a Zorbax Eclipse Plus C18 column (100 × 2.1 mm i.d., 1.8 μm particle size; Agilent Technologies). The mobile phase consisted of 5 mM ammonium formate in 0.02 % formic acid (solvent A) and a mixture of acetonitrile/water (90:10, v/v) containing 5 mM ammonium formate and 0.02 % formic acid (solvent B). A gradient elution program was employed at a constant flow rate of 0.2 mL/min with solvent B starting at 5 % for 3 min, initially increasing to 30 % in 4 min, then increasing to 90 % in 11 min and finally, set back to 5 % in 11.5 min. Post-run equilibrium time was 3.5 min. The injection volume was 3 μL. A QExactive benchtop Orbitrap-based mass spectrometer (ThermoScientific, Bremen, Germany) equipped with a heated electro-spray ionization (HESI) source operated in negative polarity mode in full scan from *m/z* 100–1000 at 17,500 resolving power and injection time of 100 ms. Source parameters were: sheath gas (nitrogen) flow rate, auxiliary gas (nitrogen) flow rate and sweep gas flow rate: 40, 10 and 1 arbitrary units respectively, capillary temperature: 300 °C, ESI heater temperature: 30 °C, spray voltage: +4.0 kV. For the structural investigation of metabolites LC-HRMS product ions scans were performed for the deprotonated molecules at selected ions with an isolation window of *m/z* 1.0 at 17,500 resolving power in various collision energies and injection time of 100 ms (product ion mode). The automatic gain control (AGC) was set at 10E6 ions. The mass calibration of the Orbitrap instrument was evaluated in both positive and negative modes weekly and external calibration was performed prior to use following the manufacturer’s calibration protocol.

**4.2 Laboratory 2**

The LC-HRMS/MS analysis was performed using a Vanquish UHPLC system coupled via a HESI source to an Orbitrap Exploris 480 mass spectrometer (ThermoFisher Scientific). The chromatographic separation was carried out using an EC 4/2 Nucleodur C-18 Pyramid 3 μm (4 × 2 mm) pre-column and an EC 50/2 Nucleodur C-18 Pyramid 1.8 μm (50×2 mm) analytical column (Macherey–Nagel). The mobile phase was a 5nmol ammonium acetate buffer containing 0.1 % acetic acid (pH 3.5), as solvent A, and ACN was used as solvent B. The LC gradient had a total run time of 16.5 min and was set as follows: starting conditions 25 % B and flow rate of 200 µL/min, increasing in 5 min to 40 % B and in further 6 min to 100 % B. The 100 % B was maintained for 1 min at 200 µL/min and 1 min at 350 µL/min followed by re-equilibration at 25 % B for 3 min at 350 µL/min and 0.5 min at 200 µL/min. The MS was operated in negative ionization mode with an ionization voltage of 3000 V and a transfer tube temperature of 350 °C. The full scan experiments were conducted with a resolution of 60,000 full width at half maximum (FWHM) and a range of *m/z* 100–800, while the MS/MS experiments were conducted in single ion monitoring (SIM) for *m/z* 393.0679 and *m/z* 186.0172 mode with a resolution of 60,000 FWHM.

**5. *In Vitro* Binding & Transactivation Properties**

Human PC3(AR)2 cells were proliferated in DMEM/F12 supplemented with 1 % P/S, 0.25 mg/mL G418 and 10 % FBS. For reporter gene assays, the cells were cultivated in DMEM/F12 containing 1 % P/S and 5 % dextran-coated charcoal treated FBS. PC3(AR)_2_ cells seeded in a 24-well plate (60,000 per well) were transiently transfected with the reporter plasmid mmTVluc (0.2 µg plasmid DNA and 0.5 µL Attractene per well). All test compounds were dissolved in DMSO. Cells were either treated with 0.1 % DMSO as solvent control, 10^−9^ M DHT as positive control or different dosages of **1**, **M2c**, **M5b** and **M8**, respectively (a final concentration of 0.1 % DMSO was kept in every treatment). For antagonization of AR transactivation, cells were co-incubated with 5×10^-7^ M bicalutamide and 10^−8^ M **1**, 10^−8^ M **M2c** or 10^−5^ M **M5b**, respectively. For the experiments investigating AR-antagonistic properties of **M8**, different dosages of **M8** were co-incubated either with 5×10^−11^ M DHT or 10^−9^ M **1**. Luciferase activity was measured according to the manufacturer’s protocol, and protein concentration was quantified using the bicinchoninic acid assay with BSA as standard protein. Relative Luminescence Units (RLU) were calculated by normalizing the luminescence with protein concentration. Three independent cell culture experiments were performed from which means ± standard deviation are shown. Statistical significance was assessed by one-way ANOVA followed by Bonferroni’s post hoc test, p < 0.05 was denoted as statistically significant.

**6. Structure Preparation & Molecular Docking**

The human androgen receptor (AR) (PDB: 5V8Q) [3] co-crystallized with 4-[(2S,3S)-2-ethyl-3-hydroxy-5-oxopyrrolidin-1-yl]-2-(trifluoromethyl)benzonitrile (SARM-2f) was used as a template for molecular docking. The raw PDB-structure was first prepared in MOE (v2022.02 Chemical Computing Group ULC, 910-1010 Sherbrooke St. W., Montreal, QC H3A 2R7, 2024) by building missing loops, adding missing atoms, and capping chain ends. Ionization states in the protein were then assigned, by protonating the PDB-structure at pH 7.4 using the Protonate3D algorithm [4].

Ligands were then built in LigandScout (v4.4.3) [5]. Because of the carboxylic acid in **M5b**, molecular docking was performed for both the protonated and deprotonated state. Additionally, ligands were built for both stereoisomers of **M8**. Then, ten binding hypotheses were built in GOLD (v5.8.1; Genetic Optimization for Ligand Docking, The Cambridge Crystallographic Data Center, UK) [6] during the molecular docking step. All residues within a 6 Å radius of SARM-2f were defined as the binding site. After docking, binding modes were minimized using the MMFF94 force field [7] and visually inspected. For each ligand, one binding mode was selected. Binding modes that contain hydrogen bonds with Arg705 and Asn752, while maintaining favorable shape similarity with SARM-2f, were favored.

**References**

1. Fragkaki AG, Sakellariou P, Kiousi P, Kioukia-Fougia N, Tsivou M, Petrou M, et al. Human in vivo metabolism study of LGD-4033. *Drug Test Anal*. 2018;10(11–12):1635-1645. doi:10.1002/dta.2512.
2. Wagener F, Guddat S, Görgens C, Angelis YS, Petrou M, Lagojda A, et al. Investigations into the elimination profiles and metabolite ratios of micro-dosed selective androgen receptor modulator LGD-4033 for doping control purposes. *Anal Bioanal Chem*. 2022;414:225-236. doi:10.1007/s00216-021-03740-7.
3. Aikawa K, Asano M, Ono K, et al. Synthesis and biological evaluation of novel selective androgen receptor modulators (SARMs). Part III: Discovery of 4-(5-oxopyrrolidine-1-yl)benzonitrile derivative 2f as a clinical candidate. *Bioorg Med Chem*. 2017;25(13):3330-3349. doi:10.1016/j.bmc.2017.04.018.
4. Labute P. Protonate3D: Assignment of ionization states and hydrogen coordinates to macromolecular structures. *Proteins*. 2009;75(1):187-205. doi:10.1002/prot.22234.
5. Wolber G, Langer T. LigandScout: 3-D pharmacophores derived from protein-bound ligands and their use as virtual screening filters. *J Chem Inf Model*. 2005;45(1):160-169. doi:10.1021/ci049885e.
6. Jones G, Willett P, Glen RC, Leach AR, et al. Development and validation of a genetic algorithm for flexible docking. *J Mol Biol*. 1997;267(3):727-748. doi:10.1006/jmbi.1996.0897.
7. Halgren TA. Merck molecular force field. I. Basis, form, scope, parameterization, and performance of MMFF94. *J Comput Chem*. 1996;17(5-6):490-519. doi:10.1002/(SICI)1096-987X(199604)17:5/6<490::AID-JCC1>3.0.CO;2-P.
